# Improvement in the Safety of Use of Hand Dishwashing Liquids Through the Addition of Hydrophobic Plant Extracts

**DOI:** 10.1007/s11743-016-1868-x

**Published:** 2016-09-01

**Authors:** Tomasz Wasilewski, Artur Seweryn, Maciej Krajewski

**Affiliations:** Department of Chemistry, Kazimierz Pulaski University of Technology and Humanities, ul. Boleslawa Chrobrego 27, Radom, 26-600 Poland

**Keywords:** Hand dishwashing liquid, Supercritical CO_2_*Chamomilla recutita * extracts, Safety, Quality, Anti-irritant properties

## Abstract

We investigated the effect of hydrophobic extract concentration on the safety of using hand dishwashing liquids (HDL). A series of formulations was prepared, differing in the concentration of the hydrophobic chamomile extract obtained in supercritical CO_2_ conditions (from 0 to 0.7 %). We found that an increase in the concentration of the extract led to a decrease in the zein number, and reduced changes in the pH level of bovine serum albumin solution (i.e., two parameters determining the irritant activity of the formulations). It was also found that the additives reduced transepidermal water loss and improved the skin hydration level. Based on the findings of the study, a mechanism has been proposed, according to which hydrophobic plant extracts form aggregates in the volume phase of the washing bath. The surface of the aggregates is the adsorption area for surfactant monomers responsible for the irritant effect. Increasing the addition of the extract was shown to reduce the negative impact of the formulations on the skin of the hands, thus contributing to a greater safety of use of HDL.

## Introduction

Hand dishwashing liquids (HDL) are one of the most important groups of household chemicals. In physicochemical terms, they are aqueous solutions of surface-active agents and various additives including fragrances, preservatives, antioxidants, sequestrants, pH regulators, colorants, opacifiers as well as substances giving formulations a pearly appearance [1, 2]. The key factors determining the quality of HDL include the type and concentrations of basic surfactants used in the formulation. The most common are anionic surfactants from the groups of alkyl sulfates and alkyl ether sulfates [1–6].

In line with the most recent trends, hand dishwashing liquids should not only have a good functionality (appropriate detergent, foaming or rheological properties), but also demonstrate a high level of safety for the skin of the hands. Surface-active agents contained in the formulation may interact with the skin on the hands, causing skin drying and irritations [1–5].

Studies on the safety of product use published up to the present place a special emphasis on the assessment of negative effects on the skin of the hands induced by model surfactants, particularly sodium lauryl sulfate (SLS) [7–17]. It has been determined that surfactants interacting with the stratum corneum can cause tissue swelling, wash away water-soluble small molecular components of the stratum corneum, and inactivate enzymes [7–9, 12–17]. Based on scientific studies in the area, it has been suggested that the main factor contributing to the negative effect of surfactants on the skin of the hands is the presence of free surfactant monomers in the washing bath [9, 10, 12–17]. Such molecules are able to interact relatively easily with proteins found in the skin, causing undesirable skin irritations. The most frequent effect is a decrease in the skin’s barrier function leading to excessive drying. As a result, the skin becomes rough, liable to cracking or becomes inflamed [9, 10, 12, 15, 17].

Studies by Moore *et al*. contradicted the widely accepted view that only submicellar species penetrated into the skin, according to the monomer penetration model. They found that both the monomers and micelles were able to penetrate into the epidermis [7]. One of the potential means of preventing these adverse effects is the addition of co-surfactants [18–27], hydrolyzed proteins [28–30] or polymers [30–32]. The action mechanisms of the additives involve an increase in micelle size or formation of appropriate complexes leading to a decreased concentration of free surfactant molecules in the solution [15, 33].

Studies conducted to date have mainly focused on model surfactant systems. However, the majority of developed and marketed formulations represent mixtures of about a dozen different compounds. An especially important role in such formulations is played by hydrophobic substances, most commonly in the form of fragrances or plant extracts [1]. Substances of this type undergo intramicellar solubilization, leading to an increase in the size of aggregates formed in this way [34, 35]. The present study is an attempt to determine how the presence of hydrophobic ingredients in hand dishwashing liquids affects user safety.

## Materials and Methods

### Materials

Raw materials used in the commercial household products industry were used to develop HDL: sodium laureth sulfate (Texapon NSO; BASF, Ludwigshafen, Germany, cocamidopropyl betaine (Dehyton K; BASF, Ludwigshafen, Germany), Laureth-7 (PCC Rokita SA, Brzeg Dolny, Poland), PEG-40 hydrogenated castor oil (Emulsionante ELH40, Erca Poland), *Chamomilla recutita* extract—extract obtained at supercritical conditions of CO_2_ (New Chemical Syntheses Institute, Puławy, Poland), sodium chloride (NaCl; POCH, Gliwice, Poland), methylchloroisothiazolinone and methylisothiazolinone as preservatives (Euxyl K120; Schulke & Mayr, Fairfield, NJ), distilled water.

### Methods

#### Viscosity Measurements

The rheological properties of hand dishwashing liquids were evaluated based on our own methodology. A PVS Brookfield rheometer was used for measurements. Viscosity values (*η*) at various shear rates (*γ*) were determined.

Fluid samples of 23 cm^3^ were placed in the measuring cylinder (part of the apparatus). Then, the cylinder was attached to the apparatus (spindle marked B1 was used). Measurements were carried out at a temperature of 22 °C and performed using the RheoVision program, which allows for automatic measurement of the viscosity with the given shear rate, and records the results. Viscosity measurements were made at 0.1 s^−1^ (in the range of 0.1–1 s^−1^) at 1 s^−1^ intervals (in the range of 1–10 s^−1^), 10 s^−1^ (in the range of 10–100 s^−1^) and at 100 s^−1^ (in the range of 100–800 s^−1^). The viscosity values in the graphs represent the average of three independent measurements.

On the basis of the obtained results, dynamic viscosity as a function of electrolyte concentration was determined at shear rates (10 s^−1^).

#### Determination of Turbidity

The test was performed using a turbidity analyzer (turbidimeter) HACH 2100 AN. Hand dishwashing liquid was transferred to the cuvette which was then placed in the measuring chamber of the turbidimeter. The results were evaluated after their stabilization. The final result was the arithmetic mean of three independent measurements.

#### Measurement of Particle Size Distributions

The particle size distribution of hand dishwashing liquids was determined by the dynamic light scattering (DLS) technique using a Zetasizer Nano (Malvern) instrument. The size of the particles was measured 24 h after the beginning of the production. The measurements were carried out in the range between 1 and 10,000 nm, at a scattering angle of 137° and temperature of 25 °C. The presented values are the average from three runs with at least ten measurements.

#### Determination of Irritant Potential—Zein Value (ZV)

Irritant potential of the products was measured using the Zein test. In the surfactants solution zein protein is denatured and then is solubilized in the solution. This process simulates the behavior of surfactants in relation to the skin proteins [36, 37].

In the zein test procedure, 2 g of protein is solubilized in a 40 g solution of dishwashing formulation sample (10 wt%). The amount of solubilized protein was determined by Kjeldahl analysis, and the result of the zein number procedure was expressed as mg of solubilized protein in 100 mL of sample. The final result was the arithmetic mean of three independent measurements.

#### Determination of Irritant Potential–pH Rise Test with Bovine Serum Albumin (BSA)

First, 50 g of aqueous solution of BSA (2 wt%, pH = 5.5) was mixed with 50 g of HDL solution (10 wt%, pH = 5.5). The pH of the BSA and analyzed samples solution was regulated with sodium hydroxide or citric acid solutions. Samples were stirred (200 rpm, 3 h). After 72 h of incubation at room temperature, the pH was measured (pH-meter Elmetron, 22 °C). The final result was the arithmetic mean of five independent measurements. The result was presented as the difference in the pH of the solution after incubation and the pH of the solution before incubation (value 5.5) [38, 39].

#### Evaluation of the Degree of Skin Dryness After Washing

The skin drying effect triggered by the liquids under study was evaluated on the basis of a specially designed original methodology. The methodology was designed according to the recommendations of the apparatus manufacturer (Courage–Khazaka). The tests were conducted using a Corneometer CM 825 probe from Courage–Khazaka (Köln, Germany) connected to a Cutometer MPA 580 adapter. The test consisted of measuring the hydration of selected skin areas (test field and control field), performing a model washing process in such a way as to ensure that the test field remained in contact with the washing solution (the control field was washed with distilled water), followed by another measurement of skin hydration in the test and control areas after a precisely defined time. Changes in the skin hydration level (expressed as a percentage) were the basis for determining the skin drying effect of the product under study. It was assumed that the skin drying effect (expressed as percent relative to the baseline) corresponded to the scale of decrease in skin hydration after time *t* from product application, expressed as percent. The drying effect was calculated by subtracting the percent change in the hydration level of the test area from the percent change in the hydration level of the control area (after the same time period).

The measurement procedure was as follows. Prior to the application of products, squares measuring 2 cm × 2 cm (1 test field + 1 control field) were marked on the forearm skin of testers. The hydration level was determined in each field by applying the corneometer probe to the skin and reading out the result. Next, a 0.5-g portion of the test product was applied to the test area. The product was rubbed slightly and rinsed with distilled water at a temperature of 40 °C (after 5 min). The same procedure was also used in the control field, except that no product was applied. As the next step, after 2 and 3 h, corneometer measurements were repeated. Each skin section (20 × 20 mm) to which the product was applied was subjected to five measurements, 1 and 2 h after product application to the skin. Two extreme results were discarded, and an average was calculated from the remaining results.

Each of the hand dishwashing liquids was assessed on the basis of tests performed with ten testers. Before the test began, each tester received instruction describing unacceptable procedures (washing or wiping the test and control areas, physical activity, etc.).

The skin drying effect (changes in skin hydration after time *t* from product application) was calculated with the formula below by averaging the results obtained for all testers participating in the study:

$${\text{DE}} = \left( {\frac{{(N_{{{\text{K}}1}} - N_{{{\text{K}}0}} )}}{{N_{{{\text{K}}0}} }} - \frac{{(N_{{{\text{B}}1}} - N_{{{\text{B}}0}} )}}{{N_{{{\text{B}}0}} }}} \right) \times 100\,\,{\text{\%,}}$$

where DE is the skin drying effect (change in skin hydration), %, *N*_K1_ is the mean skin hydration after time *t* from model skin washing in the presence of distilled water (control field), [a.u.], *N*_K0_ is the mean skin hydration in the test area before skin washing in the presence of distilled water (control field), [a.u.], *N*_B1_ is the mean skin hydration after time *t* from model skin washing in the presence of the test product (test field), [a.u.], *N*_B0_ is the mean skin hydration in the test area before model skin washing (test field) [a.u.].

#### Measurement of Transepidermal Water Loss

The extent of damage to the epidermal barrier was assessed by determining TEWL (transepidermal water loss) which is a commonly accepted and a sensitive indicator of disruptions in the epidermal lipid barrier. The tests were conducted using a Tewameter TM 300 from Courage–Khazaka (Köln, Germany) connected to a Cutometer MPA 580 adapter. The Tewameter head is equipped with two pairs of sensors (temperature and moisture), and measures the gradient between them.

The measurement procedure was as follows. Prior to the application of products, squares measuring 2 cm × 2 cm (1 test field) were marked on the forearm skin of testers. Next, a 0.5-g portion of the test product was applied to the test area. The product was rubbed slightly and rinsed with distilled water at a temperature of 40 °C (after 5 min). As the next step, after 4 h, Tewameter measurements were performed. Each skin section (20 × 20 mm) to which the product was applied was subjected to ten measurements. Two extreme results were discarded, and an average was calculated from the remaining results.

Each of the hand dishwashing liquids was assessed on the basis of tests performed with 10 testers. Before the test began, each tester received instructions describing unacceptable procedures (washing or wiping the test and control areas, physical activity, etc.). The test was performed between 11:00 am and 1:00 pm to exclude potential variation over the 24-h period. The measurements were performed after a 10-min acclimatization period.

The final result was the arithmetic mean of ten values of the test parameter obtained for the skin area to which a given product was applied.

#### Statistical Analysis

The points in the charts represent mean values from a series of three or five independent measurements. The Student’s *t*-distribution was used to calculate confidence limits for the mean values. Confidence intervals, which constitute a measuring error were determined for the confidence level of 0.90. Error values are presented in the figures.

## Results and Discussion

### Development of Formulations and Technologies for Producing HDL Containing Hydrophobic Extracts

Based on the literature and our own experience [1–6], starting formulations were developed to produce HDL containing a hydrophobic plant extract. Next, the effect of additive concentration on product properties associated with the safety of use was assessed.

The additive selected for the study was a chamomile extract obtained in supercritical CO_2_ conditions. A great advantage of this type of extraction is the fact that the process takes place at a relatively low temperature. Consequently, the majority of valuable active substances can be retained in the extracts. The solvent used (CO_2_) is non-toxic, relatively easily available and cheap, and can be removed from the material on completion of the process. As a result, the process of producing the material is beneficial both in economic and environmental aspects [40–44].

Extracts obtained in supercritical CO_2_ conditions are strongly hydrophobic materials. The literature on the topic shows that they are a valuable source of multiple active ingredients such as polyunsaturated fatty acids, phytosterols, polyphenols and natural colorants [40–46]. An analysis of the qualitative composition of supercritical extracts reveals that one of the most potentially interesting ingredients from the viewpoint of application in HDL is chamomile extract [45, 46]. The composition of the extract used in the presented studies was analyzed by Michorczyk *et al*. [45], who showed that chamomile extract contains a number of substances including fatty acid triglycerides (linoleic acid—73.3 %, palmitic acid—0.8 %, oleic acid—7.7 %, α-linolenic acid—1.6 % and γ-linolenic acid—0.5 %) as well as terpene compounds and terpenoids (bisabolol oxide B—7.4 %, bisabolol oxide A—4.8 % and chamazulene—3.6 %).

The starting formulations for producing HDL prototypes contained: sodium laureth sulfate—11 %, cocamidopropyl Betaine 2 %, 1 % Laureth-7, 1 % PEG-40 hydrogenated castor oil, a mixture of methylchloroisothiazolinone and methylisothiazolinone as a preservative, and made up to 100 % with water.

A total of seven formulations were prepared, differing in the content of chamomile extract which ranged from 0.001 to 0.9 wt%. A formulation with a similar composition, but not containing any plant extract, was used as a reference.

Preparing the extract-enriched formulations required an appropriate procedure. In the first stage, the chamomile extract was mixed with PEG-40 hydrogenated castor oil, so as to enable the hydrophobic extract to be thoroughly solubilized at a later stage. The two ingredients were mixed at a temperature of 40 °C. The remaining ingredients were mixed with water at room temperature. Next, the mixture of the plant extract and solubilizer was added to the system and the entire contents were mixed thoroughly.

The formulations obtained at this stage were clear and stable. The next stage, aimed at obtaining a prototypical HDL, was the selection of an appropriate amount of a viscosity modifier. An assumption was made that the prepared formulations would have a similar viscosity, varying around 1000 mPa·s. Sodium chloride was used as a viscosity modifier in the system [34].

Based on the compositions thus designed, seven formulation batches were prepared, the variable parameter being the concentration of sodium chloride (tests were performed for sodium chloride concentrations ranging from 0 to 3 %). Adding an appropriate weight of salt to the formulation resulted in a corresponding decrease in water content. The liquids were produced according to the procedure described above. Sodium chloride was added in the final stage, following which the entire contents were mixed until the dissolution of salt. Then, the correlations between viscosity and sodium chloride concentration were determined for formulations containing various chamomile extract concentrations.

The determined correlations were used as a basis for estimating the amount of sodium chloride that allows one to obtain HDL with a viscosity close to 1000 mPa·s. The compositions of prototypical HDL with different contents of the hydrophobic plant extract are listed in Table 1.

**Table 1:** Model formulations of hand dishwashing liquids

Component (INCI name)	Concentration (wt%)							
	0E	0.001E	0.01E	0.1E	0.3E	0.5E	0.7E	0.9E
Sodium laureth sulfate	11							
Cocamidopropyl betaine	2							
Laureth-7	1							
PEG-40 hydrogenated castor oil	1							
*Chamomila recutita* CO_2_ extract	0	0.001	0.01	0.1	0.3	0.5	0.7	0.9
Sodium chloride	1.4	1.5	1.5	1.5	1.5	1.4	1.4	1.3
Methylchloroisothiazolinone and methylisothiazolinone	0.1–100							
Aqua								

**Fig. 1:**
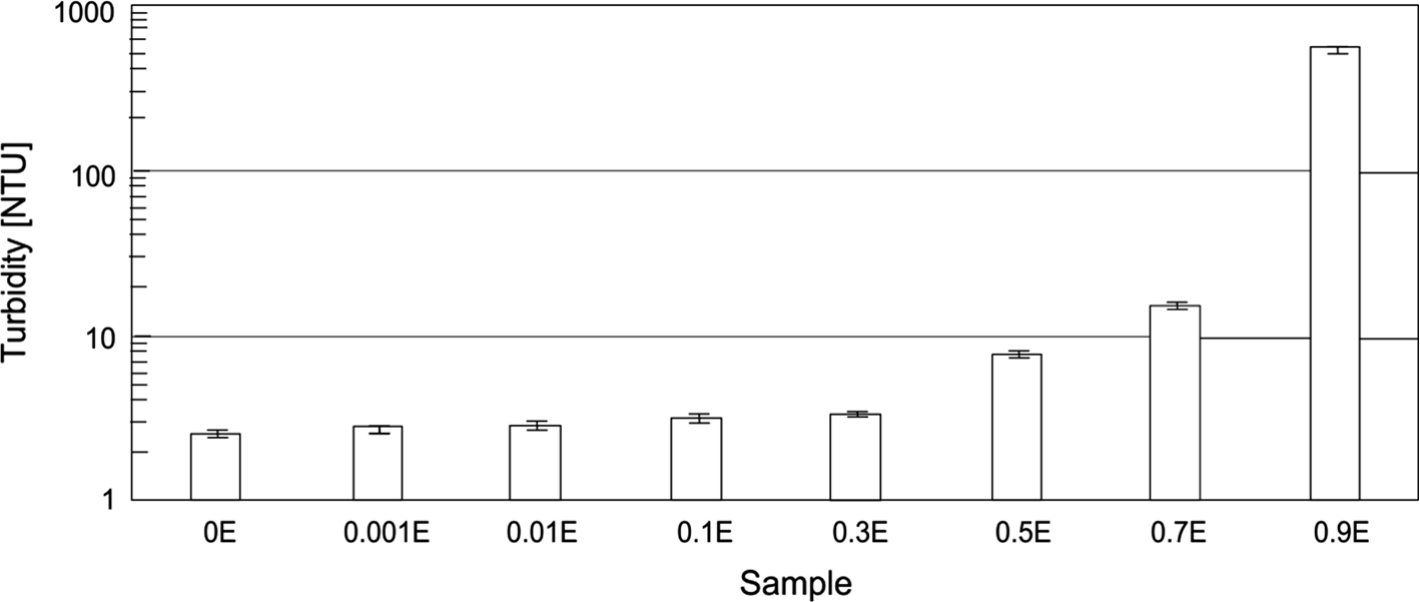
Turbidity of hand dishwashing liquids

The finished formulations were subjected to turbidity tests. The results are listed in Fig. 1.

**Fig. 2:**
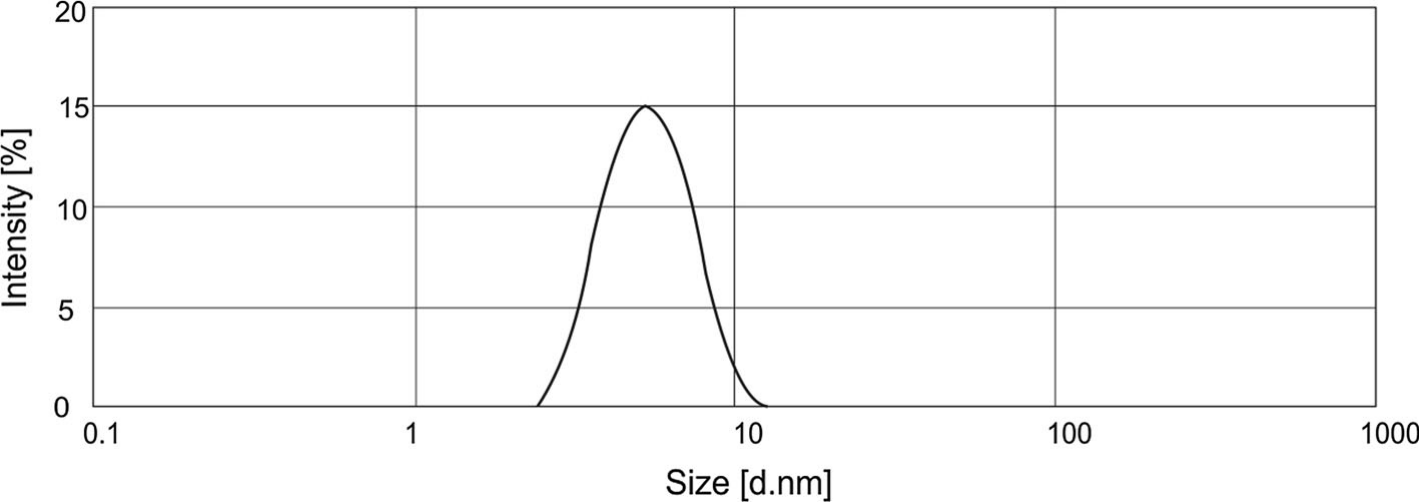
Particle size distribution for 1 % solutions of HDL without hydrophobic chamomile extract (0E)

**Fig. 3:**
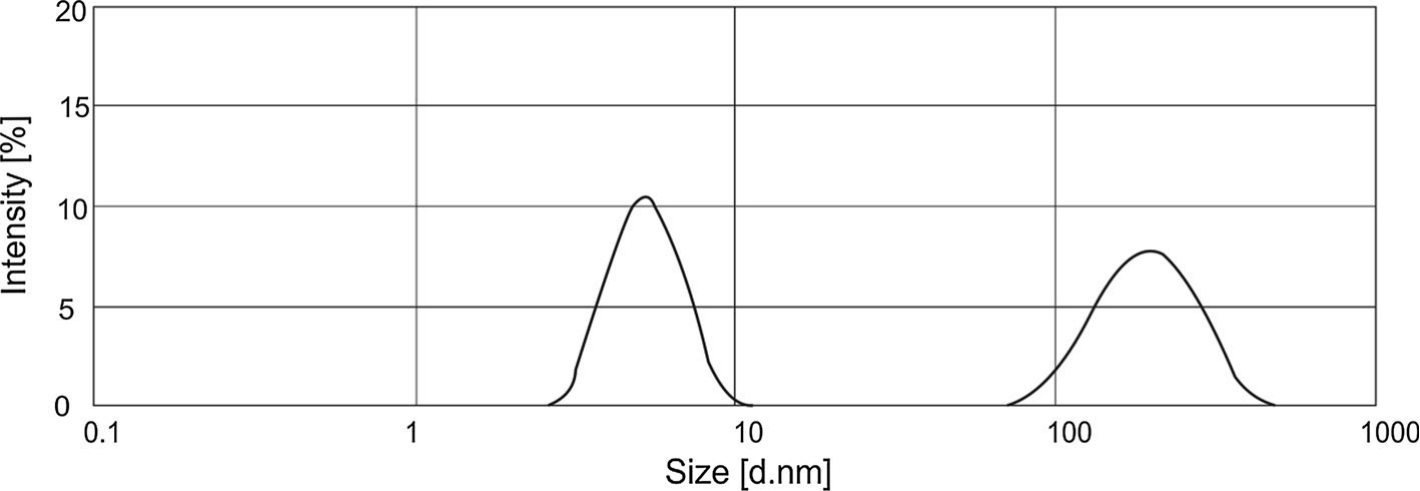
Particle size distribution for 1 % solutions of HDL with 0.7 % hydrophobic chamomile extract (0.7E)

The turbidity of the HDL fall within a broad range of 2–9.5 to 780 NTU. The turbidity obtained for the 0.3 % concentration of the chamomile extract was 5 NTU. A significant increase in the turbidity was noted at higher extract concentrations—the highest value (780 NTU) was obtained for a 0.9 % content of the plant-based material in the liquid. The formulation is not transparent, and since its turbidity markedly exceeds levels found in commercially available products, it was excluded from further tests. The turbidity increases with the concentration of the hydrophobic phase in the sample. The solubilization of the chamomile extract in micelles induces an increase in their size. The resulting aggregates are significantly larger than the micelles formed by surfactants. This leads to a rise in the turbidity of the formulations. For verification purposes, an analysis of particle size distribution was carried out for 1 % aqueous solutions of the formulations. Figures 2 and 3 show the results of the test performed for liquid formulations not containing any extract and with a 0.7 % content of the hydrophobic chamomile extract.

The results obtained for the base formulation reveal one characteristic peak corresponding to the presence of particles in the diameter range of 3–11 nm. The observed particles of this size most likely represent micelles which form above the surfactant critical micelle concentration (CMC). In the formulation containing the hydrophobic chamomile extract, a decrease in intensity was noted for particles in the size range of 3–11 nm, coupled with the emergence of a peak for particles with a size range of 80–600 nm. Large-sized particles which are observed in the solution most likely represent aggregates arising from the process of micellar solubilization of the hydrophobic plant extract. The results are thus consistent with the results obtained in tests of solution turbidity (Fig. 1).

### Skin Irritation Potential of HDL

**Fig. 4:**
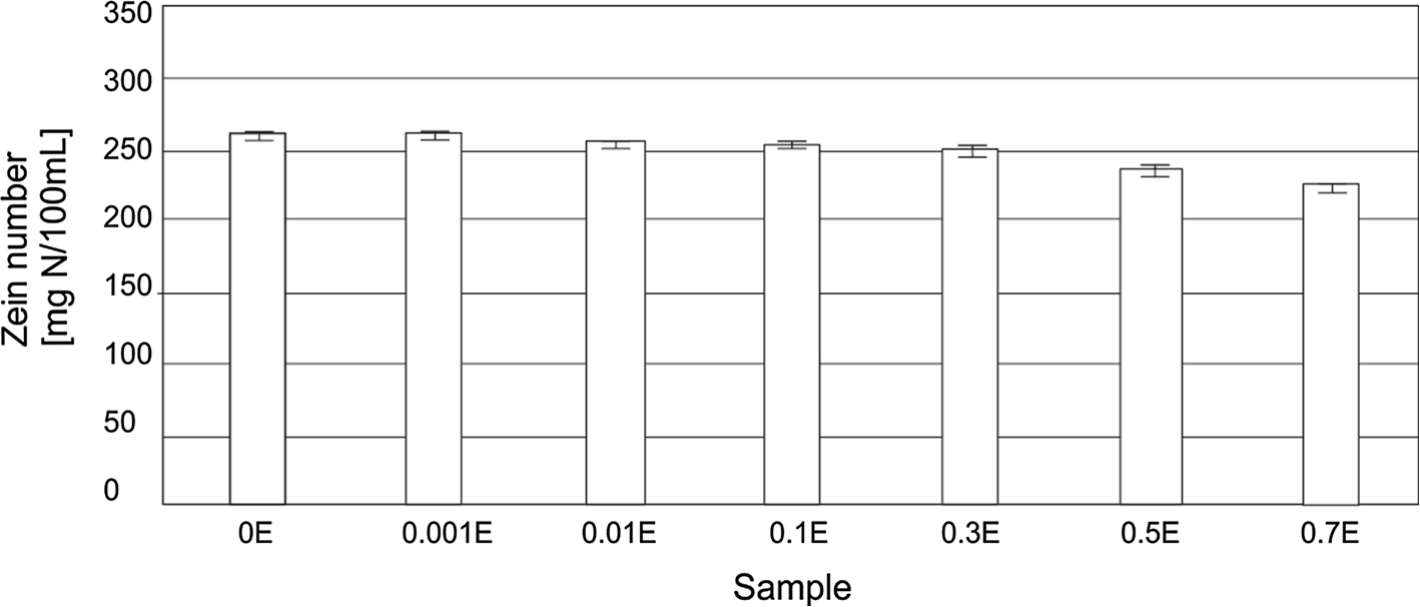
Zein numbers for 10 % solutions of hand dishwashing liquids

**Fig. 5:**
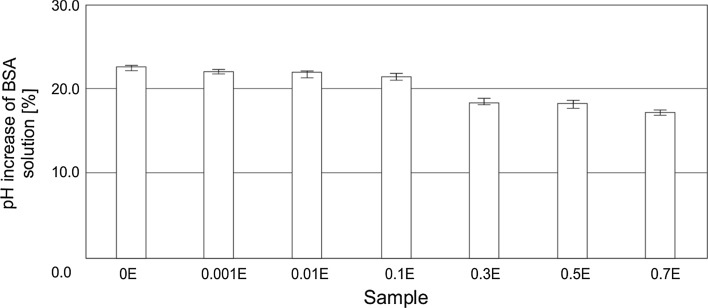
pH increase of BSA solution mixed with HDL

The safety of use, with an emphasis on the skin irritation potential of HDL, was assessed by determining the zein number [36, 37] (Fig. 4) and by measuring a change in the pH of bovine serum albumin (BSA) solution (Fig. 5) [38, 39].

#### Zein Value (ZV)

The results of zein value determinations for the studied group of products are shown in Fig. 4.

The formulations under study had zein numbers (ZN) in the range of 224–260 mg N/100 mL. The highest ZN was noted for the formulation not containing any plant extract and for the formulation with the lowest concentration used: the result was 260 units. For liquids enriched with the chamomile extract at the concentration of 0.01–0.3 wt%., there was a slight decrease in the measured parameter, however the results are within the margin of measurement error. A significant drop in ZN, amounting to 10–15 % relative to the base formulation, was found for the 0.5E and 0.7E liquids. The results indicate that the addition of a hydrophobic extract to HDL has an impact on reducing their potential to cause irritation of the skin on the hands.

The findings of the zein test were confirmed by determining the change in pH of bovine serum albumin (BSA) solution. An interaction of water-soluble BSA with anionic surfactant molecules leads to the neutralization of cationic protein groups. Consequently, protons are adsorbed from the solution by negatively charged BSA groups, resulting in a rise in the pH of the solution. The higher the pH increase, the more pronounced the skin irritating effect caused by surfactants [38, 39]. Measurement results are presented in Fig. 5.

The findings are in agreement with the results of the zein test. The highest increase in the pH value, by ca. 22.5 %, was observed for the base sample (0E). Adding an extract to the liquid formulations contributes to a decrease in the value of the parameter. A significant drop in pH was noted for formulations containing between 0.3 and 0.7 wt%. of the hydrophobic extract. Unquestionably the lowest pH increase of the BSA solution was observed for the liquid containing 0.7 wt% of the extract (0.7E). The difference in relation to the base sample was just 17.3 %. The results show that even a small addition of a hydrophobic extract, at concentrations in the range of 0.3–0.7 %, to HDL formulations reduces their skin irritation effect.

The observed relief of the irritation effect resulting from hydrophobic extract addition is consistent with the outcomes of studies conducted by Mukherjee *et al*. [47]. The authors investigated polar sunflower oil and completely non-polar mineral oil. It was found that the addition of oil resulted in a significant reduction in skin irritations, however, the activity was only observed in the case of using polar sunflower oil. The researchers attribute the effect to the fact that different oils variously interact with proteins present in the stratum corneum.

A range of studies show that the skin irritation effect is largely attributable to the presence of surfactant monomers in the washing bath [8–17]. The severity of irritations depends for the most part on the structure of the surfactant molecule and its concentration in the washing bath. A variety of additives are used to counteract the phenomenon.

For example, Bujak *et al*. [30] utilized hydrolyzed proteins and polyvinylpyrrolidone as additives to washing formulations. Based on the results obtained in the determination of the zein number and the bovine serum albumin test, the authors found drops in value exceeding 20 % relative to additive-free formulations. The action mechanism of the additives involves the formation of specific-type complexes in the volume phase of the washing bath. The complexes bind the prevailing part of free surfactant monomers [30, 33].

Also, Cohen *et al*. in their studies propose that a reduction in the skin irritation potential may be achieved by replacing commonly used anionic surfactants with sulfoxylated methyl esters [48] and alkylbenzene sulfonates (LAS) with various counterions [49]. The authors compare the results with the determined values of the critical micelle concentration (CMC) for the compounds. They show that lower values of the zein number have been recorded for compounds with lower CMC values. With respect to reducing the irritant activity of the formulations through the use of hydrophobic plant extracts, the results reported by the authors are comparable with those discussed above.

**Fig. 6:**
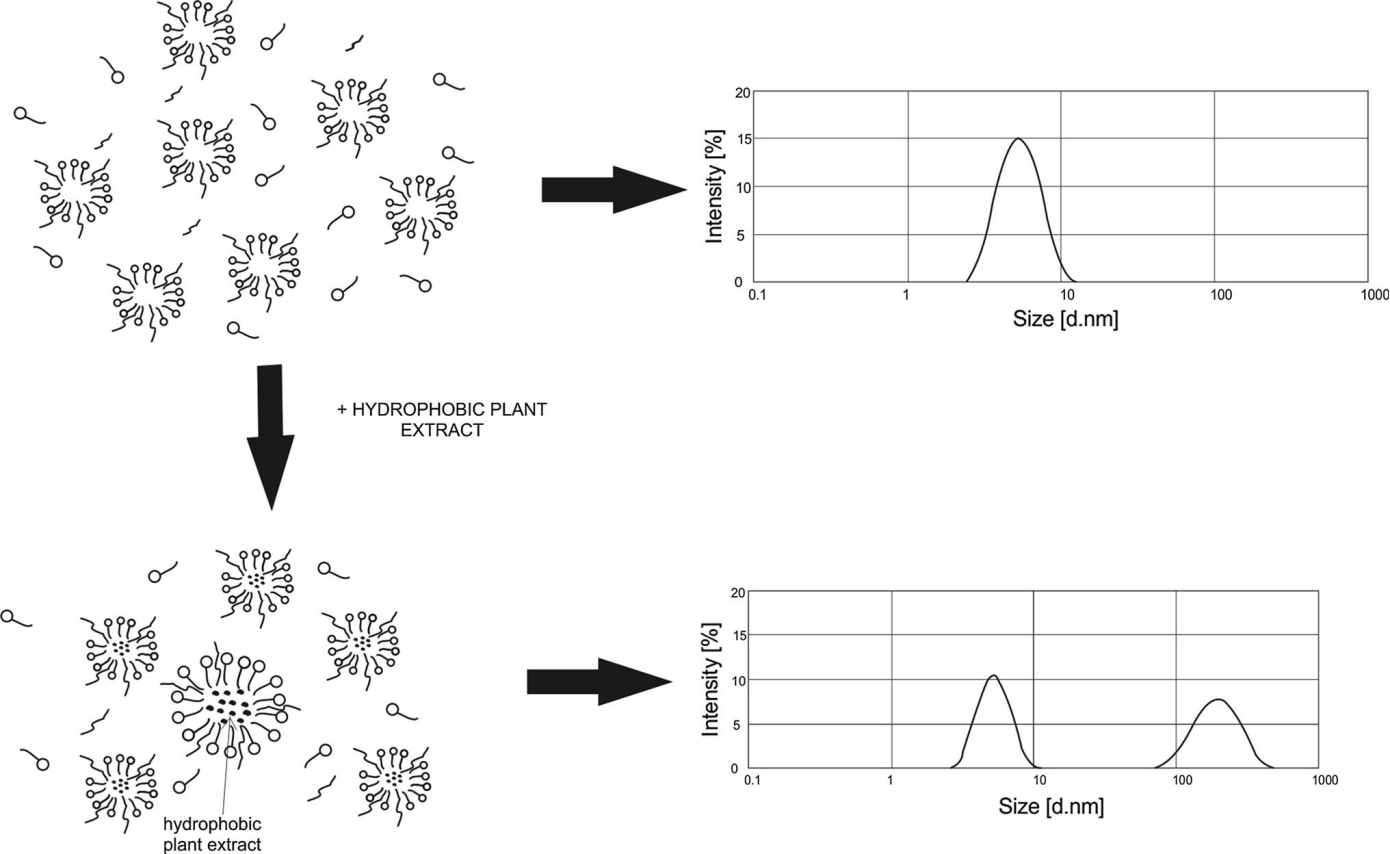
Scheme of the changes in micellar solution following the addition of hydrophobic extract

HDL typically contain a formulation of surfactants with appropriately low CMC values, so that the surfactants form micelles in the washing bath. The formation of micelles, however, does not eliminate surfactant monomers from the solution: individual molecules exist in equilibrium with micelle-forming surfactants [9, 10, 34]. In the literature [3, 5, 15, 25, 26, 29] it is postulated that an addition of amphoteric, nonionic or cationic surfactants to formulations results in a reduction in electrostatic interactions between the surfactant heads, leading to an increase in the distance between hydrophilic heads and an attenuation of their mutual repulsive forces. Consequently, the binding of the surfactant to a given micelle is stronger, and the micelles increase in size. The contact of surfactant molecules with the skin surface is thus impeded. As a result, their interactions with the skin proteins are less prominent, and the skin irritation potential is markedly reduced [15, 25, 26, 29, 31, 50, 51]. Adding a hydrophobic phase to an aqueous surfactant solution also contributes to a major change in their arrangement. A schematic representation is shown in Fig. 6.

Results demonstrating a decrease in irritant activity (Figs. 3, 4) point to the conclusion that the presence of a hydrophobic phase may lead to a decrease in the number of surfactant monomers. In the case of low hydrophobic phase concentrations, intramicellar solubilization occurs: the micelles transform into aggregates containing a hydrophobic substance, and an adsorptive surfactant layer forms on their surface. If the diameter of an aggregate formed in this manner is smaller than 100 nm, the solutions remain transparent [33, 34]. A significant factor is that the incorporation of a hydrophobic phase into a surfactant solution results in the formation of an “additional” interface (between the hydrophobic and hydrophilic phases). It may have an effect on reducing free surfactant molecules in the system. A consequence is a drop in the concentration of monomers potentially interacting with the skin and causing skin irritations.

### Evaluation of Skin Condition After the Washing Process

HDL formulations are designed with a focus on ensuring a powerful detergent activity (i.e., a high ability to remove soiling from the surface of different materials and efficacy in fat emulsification in the washing bath). However, it needs to be considered that surfactants, especially of the nonionic type, effectively reduce interfacial tension and have relatively high fat emulsification ability [1–6, 34]. Contact with the skin (during the washing process) can induce excessive elimination of lipids from the epidermis. As a result, the washing process depletes the protective layer of the skin, removing primarily valuable fatty components. If this happens, the skin is no longer a sufficient barrier against external factors, which intensifies transepidermal water loss and causes rapid skin drying.

**Fig. 7:**
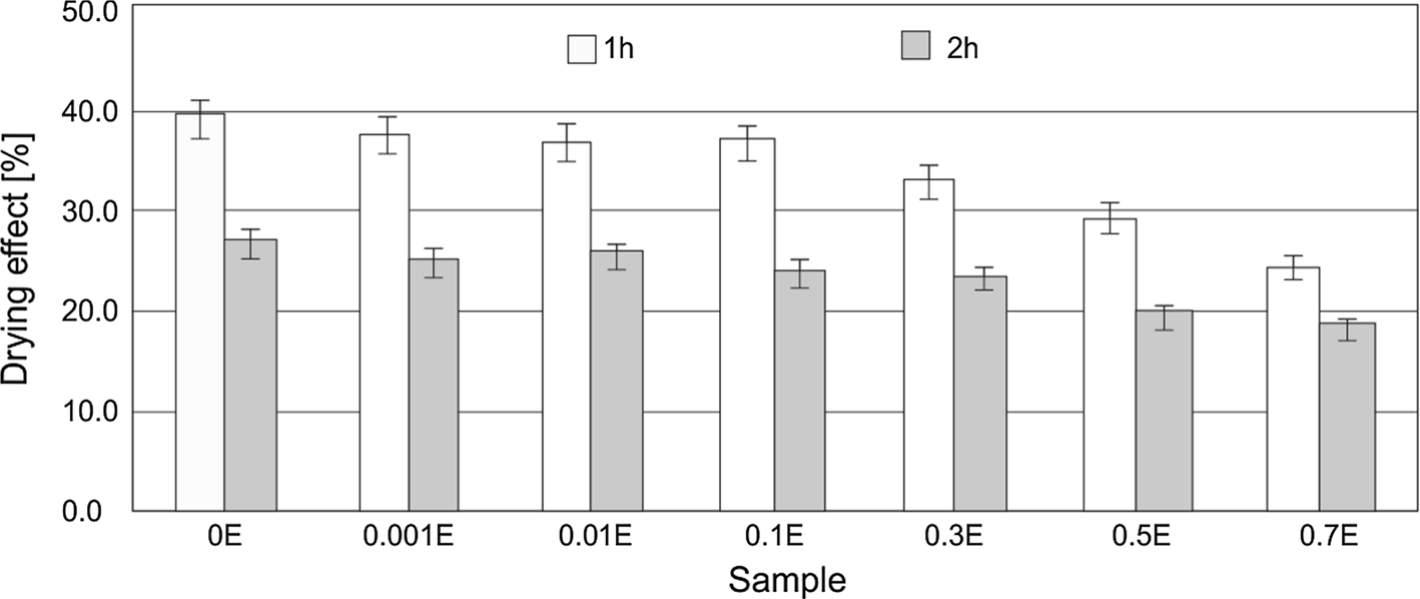
Drying effect of hand dishwashing liquids

**Fig. 8:**
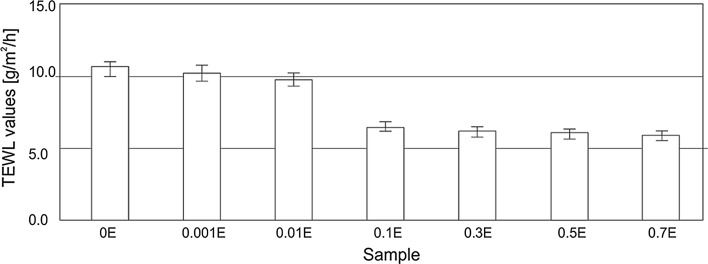
TEWL values of the skin after washing determined for the hand dishwashing liquids

Adding hydrophobic plant extracts to HDL compositions naturally restricts their detergent activity. Hydrophobic substances are usually the main components of soils, and the primary goal of using washing agents is to remove substances of this type from washed surfaces. It appears obvious that the presence of a hydrophobic ingredient in product composition will have an impact on reducing its functionality. Earlier studies conducted by the authors show that an addition of a hydrophobic plant extract at a level of up to 0.7 % indeed triggers a decrease in detergent activity, but the effect is inconsequential and has no major effect on consumer perception. It is interesting to analyze, though, to what extent extracts influence the condition of the skin. To this end, corneometer and tewameter measurements were carried out, and their results are shown in Figs. 7 and 8.

All the HDL under study exhibited a drying effect (DE) towards the skin, but the highest values (39.4 and 27 % after one and 2 h of testing, respectively) were noted for the extract-free formulation. Using a chamomile extract at relatively low concentrations (0.001–0.1 %) has no significant effect on the value of the determined parameter. It is only at higher concentrations (0.3–0.7 %) that a decrease in the determined value by nearly 10–15 % relative to the base formulation was noted.

In the series of formulations under analysis, there was a gradual drop in transepidermal water loss (TEWL) along with increasing extract concentration in the formulations. At relatively low chamomile extract concentrations, the additive was not shown to have any effect on the determined parameter. TEWL values range from 9.8 to 10.5 g/m^2^/h and are within the limits of measurement error. Markedly lower values were observed for preparations containing extracts at concentrations in the range of 0.1–0.7 %. The studied parameter was found to have decreased by nearly 40 % compared to the base formulation (0E).

It was thus proven that the use of a herbal extract at higher concentrations in a product gives an opportunity to obtain satisfying results in terms of potential prevention of epidermal damage after the washing process.

A consideration of factors associated with the quality of HDL must also involve the aspect of changing consumer expectations. An increasing number of consumers now expect a decrease in the negative effect of products on the skin of the hands. Another important aspect is the fact that nowadays many commercially available food products contain an array of substances which are, in fact, surfactants. Consequently, washing products are no longer expected to have very powerful fat-removing and cleaning properties. Instead, more balanced compositions, developed with attention to their effect on the skin of the hands, are in demand.

Chamomile extract obtained under supercritical CO_2_ conditions is a substance with hydrophobic qualities. In the washing bath, some surfactants can be used up in the solubilization process. In this manner, the concentration of surfactants being in direct contact with the skin is reduced, which has an impact on emulsification and lipid removal from the outer skin layer. It can be assumed that a hydrophobic substance present in a formulation serves as a “protective shield” for the skin of the hands. In effect, damage to the epidermal barrier preventing water evaporation from the skin is less pronounced, which is evidenced in the test results obtained for skin drying and transepidermal water loss (TEWL) after the washing process [52–56].

The chamomile extract used in HDL can have a positive skin effect not only due to its hydrophobicity but also due to the content of compounds with a proven medicinal benefit. Higher polyunsaturated fatty acids (referred to as vitamin F) and their esters have a moisturizing activity. They are helpful in the treatment of certain skin conditions including psoriasis, acne and atopic dermatitis accompanied by excessive epidermal dryness [57–60]. Bisabolol has antibacterial and antiinflammatory properties and is well-tolerated by the skin. As a result, it can be used in baby cosmetics [61]. Chamazulene, in addition to its antibacterial, antiinflammatory and antifungal effects, also has antioxidant qualities [62, 63].

## Conclusions

This study assessed the effect of the concentration of a hydrophobic chamomile extract on the properties which determine the safety of use of hand dishwashing liquids. The study yielded a number of findings:

- The presence of a hydrophobic extract in a product slightly affects the concentration of sodium chloride which is necessary for achieving optimum viscosity.
- Adding a chamomile extract at concentrations in the range of 0.3–0.7 wt% to HDL has a significant impact on the determined parameters related to the safety of product use (both in the context of irritant and drying effects). The intensity of the changes was found to increase along with increasing extract concentrations.
- The reduction in negative impact on the skin is most likely associated with the formation of particles in the volume phase of the washing bath and the formation of an “additional” interface. The process leads to a decrease in free surfactant molecules in the system, which improves the safety of use of HDL.
